# Phototherapy as a Rational Antioxidant Treatment Modality in COVID-19 Management; New Concept and Strategic Approach: Critical Review

**DOI:** 10.3390/antiox9090875

**Published:** 2020-09-16

**Authors:** Reem Hanna, Snehal Dalvi, Tudor Sălăgean, Ioana Roxana Bordea, Stefano Benedicenti

**Affiliations:** 1 Department of Surgical Sciences and Integrated Diagnostics, Laser Therapy Centre, University of Genoa, Viale Benedetto XV,6, 16132 Genoa, Italy; drsnehaldeotale@gmail.com (S.D.); stefano.benedicenti@unige.it (S.B.); 2 Department of Oral Surgery, Dental Institute, King’s College Hospital NHS Foundation Trust, London SE5 9RS, UK; 3 Department of Periodontology, Swargiya Dadasaheb Kalmegh Smruti Dental College and Hospital, Nagpur 441110, India; 4 Department of Land Measurements and Exact Sciences, University of Agricultural Sciences and Veterinary Medicine Cluj-Napoca, 400372 Cluj-Napoca, Romania; 5 Department of Oral Rehabilitation, “Iuliu Hațieganu” University of Medicine and Pharmacy Cluj-Napoca, 400012 Cluj-Napoca, Romania; roxana.bordea@ymail.com

**Keywords:** SARS-CoV-2, COVID-19, phototherapy, photobiomodulation, photodynamic therapy, low-level laser therapy, nitric oxide, antioxidant, cytokines, vaccines

## Abstract

The COVID-19 pandemic has taken the entire globe by storm. The pathogenesis of this virus has shown a cytokine storm release, which contributes to critical or severe multi-organ failure. Currently the ultimate treatment is palliative; however, many modalities have been introduced with effective or minimal outcomes. Meanwhile, enormous efforts are ongoing to produce safe vaccines and therapies. Phototherapy has a wide range of clinical applications against various maladies. This necessitates the exploration of the role of phototherapy, if any, for COVID-19. This critical review was conducted to understand COVID-19 disease and highlights the prevailing facts that link phototherapy utilisation as a potential treatment modality for SARS-CoV-2 viral infection. The results demonstrated phototherapy’s efficacy in regulating cytokines and inflammatory mediators, increasing angiogenesis and enhancing healing in chronic pulmonary inflammatory diseases. In conclusion, this review answered the following research question. Which molecular and cellular mechanisms of action of phototherapy have demonstrated great potential in enhancing the immune response and reducing host–viral interaction in COVID-19 patients? Therefore, phototherapy is a promising treatment modality, which needs to be validated further for COVID-19 by robust and rigorous randomised, double blind, placebo-controlled, clinical trials to evaluate its impartial outcomes and safety.

## 1. Highlights

The etiopathogenesis of coronavirus disease 2019 (COVID-19), cytokine release syndrome and how they contribute in multi-organ dysfunction represent prospective therapeutic targets.

The molecular and cellular activities of phototherapy and how they can regulate COVID-19 induced a cytokine storm.

Applications of photobiomodulation and photodynamic therapies are promising treatment modalities in COVID-19 management.

Exploring the role of lasers in whole inactivated virus vaccine production and as vaccine adjuvants, which requires further investigation.

## 2. Introduction

In December 2019, the World Health Organization (WHO) was alerted of a rapid and wide-spreading pneumonia of an unknown origin, which was first detected in Wuhan, the capital city of China’s Hubei province [1,2]. With the sudden exponential rise in the number of cases and thorough sample detection, WHO announced that this infection was associated with a novel coronavirus, which was named “severe acute respiratory syndrome coronavirus 2” (SARS-CoV-2) [1]. Later, the disease was denoted as “coronavirus disease 2019” (COVID-19)’ [3]. 

Over the past seven months, this viral infection has emerged as a pandemic, encroaching lives of the global population. As of 15 September 2020, there have been 29,309,546 reported confirmed cases, including 928,890 deaths [4]. As we are suffering from this fatal catastrophe at present, worldwide research is being conducted to find ways to prevent or slow the transmission of COVID-19. At this time, there are no specific vaccines or treatments for COVID-19, but there are many ongoing clinical trials assessing prospective remedies. Until a vaccine or a treatment is found, it is necessary to provide symptomatic relief to the infected patients in order to minimise their suffering. 

Laser photonic energy possesses a wide range of local as well as systemic benefits, which have been experimented and evaluated extensively over the past few decades. Phototherapy by means of photobiomodulation therapy (PBMT) and photodynamic therapy (PDT) has shown to be an effective treatment modality, which has a significant role in tissue repair and regeneration [5], wound healing [6,7], pain alleviation [8,9] and oxidant reduction [10,11].

PBMT is a noninvasive, available, cost-saving physical therapy modality with no side effects, which also delivers antioxidant and anti-inflammatory effects [12,13,14,15]. Its anti-inflammatory effects have been studied by many authors who examined the cellular signalling responsible for these effects and concluded that reduction in calcium (Ca^+2^) sensitivity may be responsible for its anti-inflammatory effect [12,15]. PBM has fundamental advantages in terms of cell proliferation and differentiation, modulating the immune responses and improving oxygenation [16,17,18,19,20]. Therefore, it can play a crucial role in regulating the cytokines storm syndrome (CSS) in COVID-19 and improve lung function, while restoring multi-organ dysfunction. It can reduce inflammatory mediators, increasing angiogenesis and enhancing healing in chronic pulmonary inflammatory diseases. Ultrashort pulsed laser is an innovation treatment modality of PBM, which selectively inactivates the virus by utilising a femtosecond pulsed laser, which has been well documented in the literature [21]. While PDT has a crucial role in reducing or totally eliminating the potential risks of transmission of coronaviruses via blood products or its derivatives, which were observed during the outbreak of SARS and MERS [22,23], it is important to note that adding a laser therapy as an adjunctive agent to the vaccine could be the future to enhance the potency of the vaccine [24].

This is why, the role of phototherapy, if any, for COVID-19 needs to be explored. In lieu of the prevailing literature, the aim of the present critical review was to evaluate the effectiveness of phototherapy in the management of COVID-19. The following objectives were determined with an intention to fulfil the aim of this review.

To understand the etiopathogenesis of COVID-19.

To address the mechanism of action of PBM in relation to COVID-19 treatment.

To recognise the mechanism of action of PDT as a possible strategy for the management of COVID-19.

This review was conducted to address the following focused research question. “Does the molecular and cellular mechanism of action of phototherapy have the potential in reducing host–viral interaction, by regulating the immune responses, in patients infected with SARS-CoV-2?” Electronic search strategies of MEDLINE (NCBI PubMed and PMC), Cochrane Central Register of Controlled Trials (CCRCT), Scopus, Science Direct, Google Scholar, EMBASE, EBSCO, and Google Scholar databases applied from August 2000 to August 2020, with an objective to identify all the relevant data, demonstrating the role and mechanism of action of phototherapy for COVID-19 patients. Additionally, a manual search of the following journals was performed; Lancet, Viruses, Virus Research, Journal of Virology, Antiviral Research, Lasers in Medical Science, Journal of Photochemistry and Photobiology B: Biology, Photodiagnosis and Photodynamic Therapy, Biophotonics, Laser Therapy and Photobiomodulation, Photomedicine and Laser Surgery. The search strategy comprised of only terms, which were related to or described the study domain and interventions. The keywords utilised for the search strategy were “COVID-19”, “SARS-CoV-2”, “phototherapy”, “photobiomodulation therapy (PBMT)”, “low-level laser therapy (LLLT)”, photodynamic therapy (PDT), nitric oxide, antioxidant, cytokines and vaccines. In order to obtain maximum information restrictions, language or publication date was not applied. All relevant evidence on this topic existing until 10th September 2020 was gathered.

## 3. Pathophysiology and Etiopathogenesis

### 3.1. The Behaviour of the Virus

It is important to capture the current understanding of SARS-CoV-2 behaviour, given the biological complexity and diversity in its pathophysiology, mechanism and interaction with the host.

#### 3.1.1. Type I Interferon (IFN-1) Signalling Pathway

Type I interferon alpha and beta (IFN-α, IFN-β) is a cytokine that is produced against viral infection. It acts as an effective innate immune response (IIR), and its downstream cascade results in regulating viral replication and induction of an effective adaptive immune response [25]. INF type I (IFN-β) is recognised by the interferon-alpha beta-receptor 1 and 2 (IFNAR 1 and 2), which are present in the plasma membrane of most cells. Upon binding of Type I INF, the IFNAR receptors activate the Janus kinases-signal transducer and activator of transcription proteins (JAK-STAT) signalling pathway, thereby stimulating the interferon-stimulating genes, which are involved in inflammation signalling and immunomodulation [26]. They interfere with viral replication and spread via several mechanisms such as a delay in the cell metabolism or cytokine secretion, which ultimately promote the activation of adaptive immunity as well as preventing viral entry or membrane infusion [25]. It was noted that cytokine induction and signalling occurs during the SARS-CoV-2 infection [25]. The Orf6 protein produced by SARS-CoV-2 inhibits the transcriptional factor such as signal transducer and activator of transcription 1 (STAT1) inside the nucleolus resulting in reduction of interferon response. Similarly, another protein “Orf3b” reduces the expression of interferon; however, in COVID-19 these two proteins are truncated and may have lost their anti-interferon function [25].

In COVID-19, IFN-β is the most relevant interferon subtype [27]. COVID-19 may diminish antiviral responses of Type I INF resulting in uncontrolled viral replication [28]. Therefore, Type I INF should be administered as soon as the virus is detected to optimise antiviral therapy and prevent adverse events. Chinese guidelines have recommended an administration of five million units of IFN-α inhalation via nebulisation in combination with ribavirin for COVID-19 therapy [29,30]. Active viral replication causes Type I IFN upregulation and influx of macrophages and neutrophils, which are the major sources of proinflammatory cytokines. COVID-19 induces delay Type I IFN and loss of viral load control in an early phase of the infection.

#### 3.1.2. Role of Mitochondrial Antiviral-Signalling Protein (MAVS) and IIR

Recent research has identified the mitochondrial protein MAVS, located on the outer membrane of the mitochondria, as a key component of an intracellular pathway, which ultimately links mitochondria to the mammalian antiviral defence system (innate immunity) [31]. This protein can activate nuclear factor kappa-light-chain-enhancer of activated B cells (NF Kappa) and interferon regulatory transcription factor (IRF3) [32,33], which are the major players in the antiviral response and inflammation as well as acting as two major effectors of IIR. [34]. Activation of this protein releases a stream of cytokines from the infected cells; this induces an immune reaction which assists in eradicating the virus-host cell [35]. In this context, MAVS is also known as “an interferon-beta promoter stimulator I (INF-β IPS-1)” [31]. It is important to highlight that the reactive oxygen species (ROS) generated during the antiviral response acts as a negative regulator at the transcriptional level where the expression of MAVS is regulated [31,36,37]. This eukaryotic organelle “mitochondria” could be considered a crucial player in future COVID-19 therapy.

#### 3.1.3. Host–Viral Interaction

**Figure 1:**
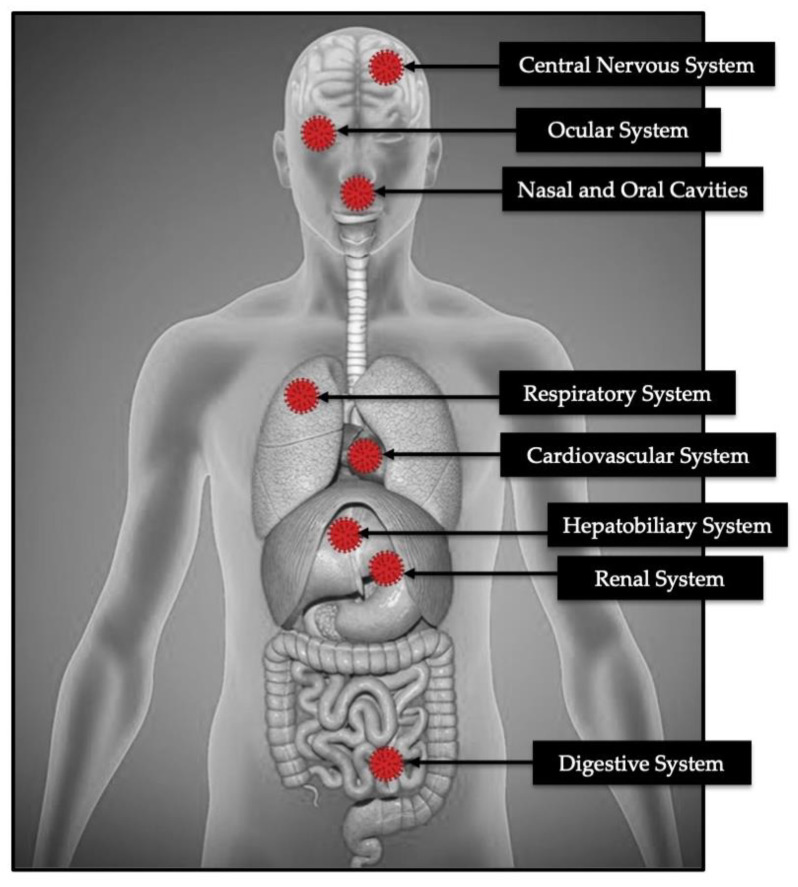
The host sites where COVID-19 spike protein binds to the ACE2.

The mechanisms underlying COVID-19 docking to the host cells are still not fully understood, especially in patients with co-morbidities [2,38]. SARS-CoV-2 enters target cells through an endosomal pathway [39]. Firstly, the receptor-binding domain of COVID-19 spike (S) protein binds to the host receptor, which is an angiotensin-converting enzyme receptor 2 (ACE2) [40], expressed in the lungs, hearts, kidneys and intestines as well as by endothelial cells [41] (Figure 1). Second, the ACE2–virus complex is then translocated to endosomes, where S protein is cleaved by the endosomal acid proteases (cathepsin L.), to activate its fusion affinity [42]. The major viral host interaction is as follows; delayed or suppressed Type I IFN response during initial infection, viral replication triggers hyper-inflammatory conditions, influx of activated neutrophils and inflammatory monocytes/macrophages, and induction of Th1/Th17 and production of specific antibodies [28].

The endothelium is a principle target of ACE2 activation and assists in minimising inflammation and ANG II-mediated vascular diseases [43]. Varga et al. (2020) demonstrated that COVID-19 is a blood vessel disease rather than what was understood initially as disease-induced pneumonia. COVID-19 attacks the ACE2 receptor of the endothelial membrane of the blood vessels leading to inflammation [44]. The ACE2 modulates angiotensin II-induced ROS production in endothelial cells. COVID-19 downregulates the ACE2 function in modulating the ANGII-induced ROS generation causing production of super oxides, ROS, which ultimately lead to build-up of the oxidative stress that initiates the disease [45].

### 3.2. Cytokines Outburst in COVID-19

Regardless of the debate in the precise concept of inflammatory outburst, the immune-mediated inflammatory responses play a crucial role in the pathogenesis of COVID-19 [46]. One of the mechanisms associated with COVID-19 progression is a significant rise in the neutrophil count and a decrease in the level of the lymphocyte count. Meanwhile, there is an increase in the levels of inflammatory markers such as ferritin, interleukin (IL) 6, Interferon-inducible protein 10 (IP-10), MCP1, tumour necrosis factor alpha (TNF-α) and D-dimer, all of which have been associated in mortality of COVID-19 and reported in various studies [47,48,49]. The main mechanism of lymphopenia in critical COVID-19 patients is still not fully understood. Nevertheless, it has been reported that a decrease in the level of B and T cells prevails in critical cases [50,51]. Additionally, it has shown that T lymphocytes trigger the spike protein of SARS-CoV-2, possibly via an endocytosis pathway, with a prominent susceptibility to the latter than SARS-CoV [52].

It is important to note that cytokine storm (CRS) is one of the main mechanisms of acute respiratory dysfunction syndrome (ARDS). A systemic inflammatory response occurs as result of release of proinflammatory cytokines storm (IFN-α, IFN-γ, IL-1β, IL-6, IL-12, IL-18, IL-33, TNF-α and TGF-β) and chemokines (CCL2, CCL3, CCL5, CXCL8, CXCL9 and CXCL10) [53]. This CRS will trigger the immune system, leading to ARDS and multiple organ failure, and subsequently death in critical cases [54].

### 3.3. The Severity of COVID-19 in Co-Relation to Biochemistry Analysis

Increased procalcitonin values are associated with a nearly fivefold higher risk of severe SARS-CoV-2 infection. As the synthesis of this biomarker is inhibited by INF-γ, whose concentration is expected to increase during viral infections, the authors of this study speculated that increased pro-calcitonin could reflect bacterial super-infection in severe disease cases. However, more investigations are needed to identify the origin of the biomarker [55]. The angiotensin II level in the plasma sample from COVID-19 patients was markedly elevated and linearly associated with viral load and lung injury [56]. Alanine aminotransferase, LDH levels, high-sensitivity CRP and ferritin were significantly higher in severe cases than moderate cases. IL-2R, TNF-α and IL-10 concentrations on admission were significantly higher in severe cases than moderate cases [57,58]. It is important to note that in SARS-CoV-2, many contributing factors play a role in the coagulation cascade turbulences, which are as follows, persistent inflammatory status, fibrinolytic activity suppression by IL-6 and induction of coagulation cascade dysfunction (hypercoagulation), due to pulmonary and peripheral endothelial injuries [47].

## 4. The Rational in Utilising Phototherapy in COVID-19 Management

Current studies and clinical trials are focused on antiviral, anti-inflammatory, cytokine syndrome suppression and increasing tissue oxygenation in the management of COVID-19 [59]. On this note, phototherapy can be instrumental in modulating the immune system and acting as an antiviral and anti-inflammatory agent.

### 4.1. Photobiomodulation (PBM) Therapy (PBMT)

PBMT is a noninvasive effective tool without any adverse effects which modulates the molecular and cellular activities for therapeutic purposes [60] such as lymphoedema [61], stroke, Alzheimer’s disease, lung inflammation, diabetic wound healing [60], tissue regeneration and chronic obstructive pulmonary disorder (COPD) [62]. Well-documented publications have shown that red and near-infrared (NIR) lights prompt tissue healing by downregulation of inflammatory cytokines and increased angiogenesis [63,64]. PBMT has been utilised in the management of viral infections by suppressing the virus replication and modulating the inflammatory cytokines [61]. Moreover, blue wavelength (λ445 nm) PBM irradiation has positive effects in reducing the viral load of HSV-1 [65]. The photonic energy of the red and NIR lights is absorbed by the cytochrome C oxidase (CCO) on the outer membrane of the mitochondria, which results in various molecular and cellular signalling cascades which are as follows, adenosine triphosphate (ATP) induction (cell proliferation and differentiation), synthesis of DNA and RNA, NO release and modification of intracellular organelle membrane activity, resulting in Ca^+2^ flux and expression of stress proteins [15,66,67,68,69].

#### 4.1.1. Mechanisms of Action and Its Relation to COVID-19

##### ATP Versus COVID-19

ATP as an intercellular signalling molecule allows modulation of molecular and cellular cascades [70], which is first observed as a result of an increase in the mitochondrial membrane potential and oxygen consumption; this results in a rapid production of NO and ROS. Subsequently, antiapoptotic proteins, antioxidant defence pathways, heat shock proteins and anti-inflammatory cytokines are increased. Cell migration and adhesion and DNA synthesis are stimulated as long-term healing parameters [71]. The biological response of vital infections to PBMT and ATP synthesis are involved in a purinergic signalling, which plays an important role in regulating the immune system [72].

Some mechanisms can explain the improvement in the muscle performance, which is due to the effects of PBMT on musculoskeletal tissues. This has already been addressed in previous studies [73,74,75]. The mechanisms that are worth highlighting are as follows, improvement in the oxidative and nitrosative stresses; an increase in the mitochondrial metabolism (CCO activity); increased haemoglobin and oxyhaemoglobin, regardless of any possible tissue heating [76]; synthesis of ATP [77]; as well as an increase in muscle glycogen synthesis and muscle cell proliferation [75]. Additionally, a recent literature demonstrated an increase in the oxygen availability in muscle cells [76,78], activation of the transcription factors, cytoprotection and protein syntheses [15].

##### NO Versus COVID-19

NO is a key part of the immune defence system. It is an endogenously produced molecule, which has a crucial defensive role against infection. NO is present in the endothelium lining of the blood vessels [79]. The white blood cells can use NO to create unstable molecules that damage the infectious pathogen. NO acts a signalling molecule and loss of its function is one of earliest indicators of a disease [80].

The proposed mechanism of action of NO is as follows. First, NO or its derivatives play a role in palmitoylation reduction expressed by S protein, which affects the ACE 2 and S protein fusion. Second, NO or its derivatives can cause a reduction in the viral RNA production at the early stage of viral replication. This is possibly due to an effect on either one or both of the cysteine proteases encoded in Orf1a (protein expression and amino acid substitutions) of SARS-CoV [81]. This was supported by Li et al. (2006), who stated that NO is effective in reducing the cell–cell fusion activity of the S protein of SARS-CoV S protein [82]. Additionally, NO inhibits the replication cycle of SARS-CoV [83]. The evidence showed that COVID-19 patients have a low level of NO (less natural production). Therefore, PBMT, which enhances the production of NO, can be utilised as an adjunctive therapy [12].

An in vivo animal study by Keyaerts et al. (2004) has shown that the virus replication reduced and cytopathic effects inhibited in SARS-CoV infected cells when treated with donor NO [84]. Currently, based on this, an ongoing clinical trial entitled “Nitric Oxide Gas Inhalation for Severe Acute Respiratory Syndrome in COVID-19”, aiming to evaluate the use of NO inhalation to improve oxygenation in moderate to high risks patients and prevent them from developing the severe outcomes of COVID-19 which require intensive care management (mechanical ventilation) [85]. However, the dose delivery is the key in optimising the clinical outcome, as a higher dose of NO can lead to toxicity.

Perhaps PBMT can be considered as a safe modality as it stimulates NO, which is a potent vasodilator by increasing the blood flow, facilitating more oxygenation to the stressed tissue as well as increasing the lymphatic flow by raising the neutrophil levels. Therefore, PBMT optimises the inflammatory processes as an anti-inflammatory and an antioxidant, which drastically improves immunity and tissue repair [12]. The release of NO into the cytoplasm and vasculature after being stimulated with PBM photonic energy prevents smooth muscle cells proliferation in the artery walls, adhesion of leukocytes and platelet aggregation, as well as reduces the oxidation of low-density lipoprotein cholesterol (LDL) (major component of plaque). This therapy can be considered to protect against atherogenesis, which is predominant in severe and high-risk cases of COVID-19 patients. Supplementary to this concept, Shi et al. (2020) highlighted that 20% of 416 patients hospitalised in Wuhan with the coronavirus had heart damage [58].

A single-blind, placebo-controlled randomised clinical trial by Mitchell at al. (2013) was conducted to measure and evaluate the effects of NIR LEDs PBM (λ 880 nm and λ 890 nm at power output of 636 mW per four pads) on venous NO, by its nitrite and nitrate metabolites, in venous blood draining from the tissue of 15 healthy young recruited subjects (21–27 years old). Positive results have shown a peak increase in the venous NO level only for five minutes into the treatment, and then slowly dropped [86]. NIR light proves to stimulate and upregulate the local production of NO through various methods of administration to act as an antiviral agent in inhibiting RNA replication of the virus, enhancing the immune system and minimising atherosclerosis in healthy young individuals. Whether this response can be achieved with patients with co-morbidities remains to be investigated.

##### ROS

ROS is a major contributor in the immune responses as signalling messengers, which are produced via nicotinamide the adenine dinucleotide phosphate hydrogen (NADPH) oxidases (NOX) pathway. They are produced in the endothelial cells. Reperfusion injury (reoxygenation injury) is directly related to the formation of ROS, endothelial cell restoration and increase in vascular permeability [87]. In acute lung injury, especially in severe COVID-19 cases, the virus downregulates ACE2 function, thus enhancing inflammation and causing vascular permeability [1].

An in vitro study by Amaroli et al. (2019) demonstrated that the photonic energy of NIR PBM can stimulate mitochondrial oxygen consumption and ATP synthesis in human endothelial cells (HEC). It is important to note that short exposure of NIR irradiation had no effect on the viability of HEC, but rather led to an increase in the rate of wound healing stimulation, which is most likely sustained via ROS-mediated stimulation of mitochondrial activity [88]. This study was supported by Góralczyk et al. (2015) in vitro study [89]. This promising therapeutic light can be useful to protect against inflammation-induced endothelial dysfunction in COVID-19 patients. A study by Fujimaki et al., 2003 showed that utilisation of λ830 nm PBMT at a fluence of 150 mW/cm^2^ induces the production of ROS by the neutrophils, which play a crucial role in inflammatory tissue repair and promote healing [90].

#### 4.1.2. PBM Effects on Modulating the Immune Response

PBMT acts as an immunomodulator, inducing antioxidant and anti-inflammatory effects [13,14,21]. Many studies have examined PBM cellular signalling and proved that a reduction in Ca^+2^ sensitivity is accountable for its anti-inflammatory effects [8,17,91]. As our findings in this critical review showed that COVID-19 dysregulates the immune response which is a feature of severe disease, the immune profile of SARS-CoV-2 associated with intensive care unit (ICU) admission is related to IL-1β and IFN gamma, which correlate with Th1 response, whereas IL-4 and IL-10 correlate with Th-2 response and IL-6 and TNF-α correlate with innate response. Several studies showed the effectiveness of utilising PBMT in modulating the pulmonary immune responses in COPD [14,21,92,93].

A study by Alves et al. (2017) showed radical molecular changes in lung tissue injury: an increase in the anti-inflammatory cytokine IL-10; an augmentation in the production of proinflammatory cytokines (IL-1β, TNF-α, IL-6 and IL-17) and chemokine (CXCL1/KC); and a decrease in the peri-bronchial density, collagen production, alveolar enlargement, P2X7 purinergic receptor expression and cell death 7AAD [92]. This study demonstrated that PBMT reduced the number of cells (Th2/Th17) in bronchoalveolar lavage (BAL). Therefore, it is worth considering PBMT in COVID-19 as a useful tool in regulating IL-4 and IL-10 which correlate with Th-2 response.

An in vivo animal study by Sergio et al. (2018) recruited Wistar rats affected by acute lung injury (ALI). The animals in the laser group were irradiated with λ808 nm PBM at a low power output setting (100 mW; 3.571 W/cm^2^; four points per lung), while the control group received sham irradiation. The results revealed an increase in the level of Bcl-2 mRNA and decrease in the caspase-3 mRNA level in the irradiated group, in comparison to the control group. This indicates that PBMT can modify the mRNA levels in the gene expression, which is associated with DNA fragmentation in alveolar inflammatory cells after lipopolysaccharide-induced ALI [94].

An in vivo animal study by Moraes et al. (2018) utilised λ660 nm PBM at a power output of 30 mW with a fluence of 3 J/cm^2^, aiming to reduce the lung inflammation [95]. The results showed a reduction in protein deposition, alveolar enlargement, proinflammatory cytokine secretion (such as; L-1β, IL-6 and TNF-α in BAL fluid) as well the expression of P2X7 receptor [95]. Studies have shown that PBMT can improve the injured musculature, by reducing the oxidative stress and inflammatory cytokines such as IL-6 and TNF-α, and increasing the IL-10 [96,97]. This was supported by an in vivo animal study conducted by de Lima et al. (2014) [98]. In the latter study, mice subjected to a mesenteric occlusion (45 minutes) were recruited, then euthanised after the clam was released, and subsequently intestinal reperfusion was carried out for 2 hours (h). The upper bronchus of the animal model was irradiated with λ 660 nm PBM for five minutes after starting reperfusion, at various fluences (1, 3, 5 and 7 J/cm^2^). The authors concluded that PBMT significantly induced an increase in the IL-10 levels in animals subjected to intestinal ischemia/reperfusion (i-I/R), highlighting the anti-inflammatory role of this therapy. Thus, it mitigates the i-I/R-induced ALI by modulating the anti-inflammatory responses, as well the proinflammatory cytokines release. Additionally, this study was the first, to prove that various laser fluences may well be effective in reducing the i-I/R-induced ALI [98].

Another study showed that PBMT reduced the neutrophils influx, myeloperoxidase (MPO) activity, cellular adhesion molecule-1 (CAM-1) mRNA expression as well as the oedema. It is important to note that PBMT reduced the ROS formation and increased the glutathione (GSH) concentration in lung from i-I/R group. This study demonstrated the effectiveness of PBMT in regulating the oxidative stress, which is notably raised in COVID-19 patients [99]. Additionally, PBMT can reduce mucus overproduction, collagen deposition and cytokine release [100,101]. Furthermore, PBMT can be utilised as an adjunctive therapy to reduce lung inflammation and enhance the immune system. A randomised controlled clinical study by Mehani et al. (2017) aimed to evaluate the effectiveness of PBMT acupuncture stimulation in comparison to the inspiration muscle training (IMT), on modulating the immune disturbances in patients with stable COPD [93]. The acupuncture points (large intestine 11, kidney meridian 27, large intestine 4, lung meridian 1 and lung meridian 7) were irradiated with λ904 nm at a peak power of 5W and with pulse width of 200 nanoseconds. Each point was irradiated for 90 seconds, twice per day, three times per week for two months. The specification of the laser pointer utilised in this study was LLL3A, GALAS, He-Ne Laser acupuncture. The results revealed a reduction in plasma IL-6 concentration associated with an increase in CD4+/CD8+ ratio in both groups, nevertheless, laser therapy’s effectiveness was superior to inspiratory muscle training. Interestingly, the levels of IL-6 and CD4+/ CD8+ were negatively correlated. In this context, studies conducted by Silva et al. (2014) and Peron et al. (2015) showed that PBMT might induce a reduction in mucus overproduction, cytokines release and collagen deposition [101,102].

#### 4.1.3. Effects of PBMT on Angiogenesis Versus COVID-19

The endothelium plays many important functions in the human body in terms of maintaining vascular homeostasis. Vascular endothelial growth factor (VEGF) is one of the key regulators of angiogenesis vascular permeability and the survival of endothelial cells. The early release of VEGF can increase pulmonary permeability, whereas a decrease in the VEGF and VEGF-receptor-1 (VEGFR-1) expressions in the lung which ultimately contributes into alveolar epithelial death [103].

PBMT, most importantly, influences the endothelial cells proliferation and secretion of angiogenic factors, which contribute into modulation of angiogenesis to improve in the management of diseases, which require blood vessels formation and repair [89,90]. PBMT can assist to restore the endothelium membrane of the affected site by COVID-19, as this viral infection contributes in inducing coagulation cascades dysfunction which ultimately leads to hypercoagulation [18]. An in vivo study by Cury et al. (2014) showed the effectiveness of λ660 nm and λ780 nm laser PBM in modulating VEGF secretion, MMP-2 activity and HIF-1α expression in a dose-dependent manner [104]. This was supported by another study, which confirmed that PBMT causes an increase in proliferation of vascular endothelial cells and decrease in VEGF and TGF-β secretion [89]. In terms of hypoxia and damage tissue, PBMT reduces the overexpression of hypoxia-inducible factor-1α (HIF-1α), TNF-α and IL-1β, and increases in the levels of VEGF, nerve growth factor (NGF) and S100 proteins in rats with chronic constriction injury [90]. Thus, utilising PBMT to enhance the angiogenesis in COVID-19 patients in Phase I and II of the disease, as a therapeutic approach, is the way forward.

A novel innovation, worthy of consideration, is the utilisation of PBMT as an intravascular laser irradiation of the body blood flow that can enhance immune responses [105]. Transdermal PBM (t-PBM) is another therapy that has beneficial effects on the endothelium and blood flow [106]. Studies have shown that PBMT improves the immune system [107,108,109]. A study conducted by Szmcyszyn et al. (2016) to evaluate the effects of t-PBM therapy on endothelial function [107]. Forty healthy young individuals (20–40 years old) were recruited and divided into two groups: laser group (*n* = 30) and control group (*n* = 10). Transdermal illumination of radial artery with λ808 nm (LED) was performed once a day for three consecutive days according to the following protocol; power output 50 mW, irradiance 1.6 W/cm^2^, energy 20 J/day and total energy 20 J. The result showed a beneficial effect of t-PBM therapy on endothelium and blood flow due to a significant increase in glutathione (GSH) levels and an extensive decrease in angiostatin concentration. However, no significant difference in the levels of the following biochemistry parameters observed; VEGF, FGF, symmetric dimethylarginine (SDMA), asymmetric dimethylarginine (ADMA) and NO pathway metabolites within 24 h after the laser irradiation. Based on this, it appeared that the latter protocol required further testing prior to utilising it in clinical human studies. Dominguez et al. (2020) suggested that an increase in super peroxide dismutase (SOD) synthesis as a result of red light PBM irradiation requires more attention from researchers [110]. SOD is an important enzyme to protect cells against mutation and replication when ROS are declined [90,111,112]. Furthermore, the same author suggested utilisation of t-PBM approach in COVID-19 management to control the CRS by irradiating the wrist level as a point of application with either visible or invisible diode lasers for 30 minutes per day for 3–5 days.

Interestingly, a study by Zhang et al. (2010) showed that after preconditioning the infarcted myocardium with λ635 nm PBM irradiation, prior to cell translation an increase in the activity of SOD activity and a decrease in the malondialdehyde (MDA) of an infarcted myocardium [113]. Additionally, preconditioning with PBMT increased the survival rate and angiogenesis and decreased the apoptotic percentage of implanted cells. This study established that PBM preconditioning therapy is a unique noninvasive approach, which might be worth considering in severe cases of COVID-19 [113].

#### 4.1.4. Light Emitted Diodes (LEDs)

Light-emitting diode (LED) is a PBM feature when the light emission is non-coherent and non-collimated. The biostimulatory effects of LEDs exert an anti-inflammatory as well as anti-fibrotic effects resulting in the release of inflammatory mediators [114,115,116] and fibroblast proliferation inhibition [117].

An in vivo animal study conducted by Brochetti et al. (2017) utilised mice induced with lung fibrosis (LF), as an animal model to evaluate the biostimulatory effects of λ660 nm LED on the development of the LF [118]. Positive results showed that LED treatment reduced the following factors: cell influx in the BAL, presence of dynamic and static elastases, collagen production and interstitial tissue thickening, increased levels of TNF-α, IL-17A, IL-6 and CXCL1/KC released by pneumocytes and fibroblasts fibrotic mice culture. The findings of this study revealed that LED can be a promising treatment modality for COVID-19 patients [118].

**Figure 2:**
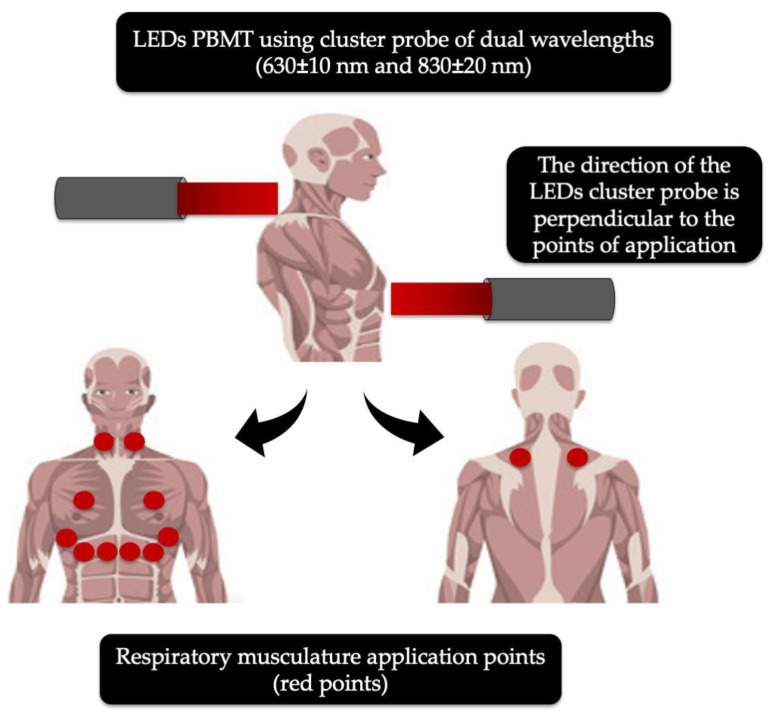
Modified schematic description of the respiratory musculature application points (red dots) for light-emitting diodes (LEDs) photobiomodulation (PBM) irradiation with 69 clustered head probe: 35 red (λ 630 ± 10 nm) and 34 of near-infrared (λ 830 ± 20 nm) in the management of COPD-19 [119].

A randomised, double-blind crossover clinical trial conducted by de Souza et al. (2019) utilised a light-emitting device of a low-intensity LEDs PBM applied on the main respiratory muscles (Figure 2) by means of a cluster with 69 LEDs, containing 35 red (λ630 ± 10 nm; 10 mW; 0.2 cm^2^) and 34 NIR (λ830 ± 20 nm; 10 mW; 0.2 cm^2^) LEDs, while the control group received sham irradiation [119]. The twelve recruited patients with COPD received two sessions of PBMT with one week apart. The primary outcomes and methods of assessment were as follows. The functional capacity assessed by the 6-min walk test (6 MWT) at baseline and 24 h after intervention, while the pulmonary function (spirometric indexes), thoracoabdominal mobility (cirtometry) and respiratory muscle strength (maximal respiratory pressures) tests were assessed at baseline, 1 h and 24 h after intervention. No significant interactions were noted for spirometric variables, maximal respiratory pressures and cirtometry. However, an increase in the functional capacity after PBMT was statistically significant (*p* < 0.01) in the 6 MWT after 24 h of innervation, compared with the baseline. The authors of this study concluded that LEDs PBMT was an effective tool for improving acute functional capacity in COPD patients observed in the 6 MWT after 24 h of intervention. Equally, this treatment can be useful to treat COVID-19 patients and is of worthy consideration, as it irradiates larger surface areas (Figure 2), as the cluster spot size is approximately 40cm^2^, which delivers two wavelengths, λ630 nm and λ830nm, with various penetration depths. Ultimately, this therapy can provide optimal therapeutic effects in COVID-19 patients [119].

A series of medical papers published over the last months, suggests that the contagion can spread deep into the vascular system and even the brain. Many studies have shown that transracial NIR PBM is a very efficient approach in reducing the cerebral ischemia both in vivo and clinical setups. The NIR PBM irradiation produces vasodilatory effects via NO-mediated pathway [120,121]. Interestingly, an in vivo animal study by Kimizuka et al. (2017) showed the effects of NIR in a mouse model of influenza vaccination using an LED device of various wavelengths: λ1061nm, λ1258 nm or λ1301 nm, which replicates the adjuvant effect of a diode pump solid-state laser (DPSSL) system [122]. The results indicated that a broad range of NIR laser wavelengths has the ability to enhance vaccine immune responses. This could be the future in utilising PBM laser therapy to enhance the vaccine properties against COVID-19.

After highlighting the mechanisms of action of PBMT in regulating and restoring the immune responses in injured tissue (oxidative stress), which are supported by in vitro and in vivo animal studies and clinically; therefore, it is important to appreciate the effectiveness of PBMT in modulating the immune responses. Ultimately, this therapy could be useful in regulating the CRS in COVID-19 patients in ICU admissions, as it enhances respiratory function and angiogenesis, therefore PBMT can be utilised in clinical studies to manage COVID-19 patients as an effective therapeutic modality at early and late phases of the infection, as well as preventive approach.

### 4.2. Photodynamic Therapy (PDT)

#### 4.2.1. Mechanism of Action of PDT

PDT is a treatment in which a photosensitiser (PS) dye is utilised in conjunction with suitable wavelength, corresponding to the absorption spectrum of the PS resulting in generation of ROS and death of the pathogen or tumour by the oxidative damage [22]. PDT induces a strong oxidative stress response in addition to triggering a vascular-mediated response, and modulating the cytokines such as IL-6 and IL-10 [123]. All these processes are sensitive to NO [124].

#### 4.2.2. Biological Response of Viral Infections to PDT

A review by Pal et al. (2020) stated that CoVs are enveloped single-stranded RNA viruses, which resemble the SARS-CoV-2 genome [125]. These viruses are generally susceptible to acid, alkaline and heat [126]. After the outbreak of SARS and MERS, many researchers investigated the potential of utilising photochemical therapy, which can reduce or totally eliminate the potential risks of transmission of coronaviruses via blood products or its derivatives [22,127,128].

The current research focuses on plasma inactivation treatments based on heat and photochemical treatment modalities. The heat method uses various temperatures and exposure times to reduce the virus concentration in the plasma. A temperature of 60 °C for 30 min exposure was found to be sufficient to reduce SARS-CoV from cell-free plasma [129], while 56 °C for 25 min exposure has reduced MERS virus by more than 4 log10 tissue culture infectious dose 50%/mL (TCID50/mL) [130]. The effectiveness of this method was due to the heat, which denatures the proteins in blood products (only manufactured plasma-derived products). On the other hand, photochemical treatment methods are based on utilising different wavelengths of light, which affect the viability of SARS and MERS viruses in the blood. Ultraviolet (UV) light, Amotosalen or riboflavin can inactivate pathogenic nucleic acids. Because the penetrating power of UV light is low the inactivation efficiency is not high enough especially when blood bags are used. Methylene blue also has a great potential in this field [131,132].

#### 4.2.3. Impact of Various PS on Viruses

##### Methylene Blue (MB)

The use of MB with visible light can inactivate coronavirus in plasma have been reported [132,133]. Eichmann et al. (2020) investigated the inactivation of three viruses, which emerged from the SARS-CoV. The results showed both THERAFLEX UV-Platelets (short-wave ultraviolet C (UVC) light) and THERAFLEX MB-Plasma (MB + visible light of λ630 nm) effectively reduce the contagion of SARS-CoV, Crimean–Congo Haemorrhagic fever virus (CCHFV) and Nipah virus (NiV) in platelet concentrates and plasma, respectively [132]. Presently, an interventional controlled clinical trial on adult COVID-19 patients is being conducted to evaluate efficiency of exchange transfusion versus plasma from convalescent patients with MB [133]. Hopefully, the results of this trial will bring some clarity to the potential role of MB in COVID-19 management. In this context, a recent in vitro study by Jin et al. (2020) concluded that BX-1 (an AIDS treatment instrument based on MB photochemistry technology) can effectively eliminate SARS-CoV-2 within 2 min and its titre decline can reach 4.5 log10 TCID50/mL [134].

##### Indocyanine Green (ICG)

ICG-based PS is a marker used to assess the perfusion of tissues and organs in many areas of medicine, especially in blood for diagnostic purposes. ICG can be activated with λ810 nm wavelength, which has a deep penetration depth. Owing to this fact, if the ICG can reach the alveolar tissue via a specific tool, it could be useful to treat COVID-19 patients [135].

##### ALA and NANO-PS

Aminolevulinic acid (ALA) and its derivatives are predominant PS in clinical PDT and have been utilised in few studies related to PDT of viruses, using haematoporphyrin derivatives [136,137,138]. Yin et al. (2012) investigated the effect of PDT using haematoporphyrin monomethyl ether (HMME) on bovine and HIV. The results showed positive responses in inhibiting the HIV in vitro experiments [139]. In terms of the nanoparticle-based approach and its effects in PDT, a study by Banerjee et al. (2012) targeted to develop ex vivo reusable antiviral agents based on protoporphyrin IX (PpIX) connected to multiwalled carbon nanotubes. The results showed a reduction in the contagion of influenza A virus in mammalian cells [140].

The above-mentioned data opens a new door for exploration of the potential of PDT in the treatment of COVID-19. Indeed, further research in this field is a necessity to claim its efficacy.

### 4.3. Ultrashort Pulsed (USP) Laser as an Antiviral Agent

The ultrashort pulsed (USP) laser is an innovative approach, as it selectively inactivates viruses by utilising femtosecond laser pulses. It has been observed that a range of fluences between 1 and 10 GW/cm^2^ allows killing of viral particles without causing cytotoxicity to the mammalian cells [141]. This process targets the intrinsic mechanical or vibrational properties of the viral capsids, which therefore become insensitive to genetic mutation of the virus, thus projecting the superiority of this technique over the current antiviral drugs. This method can be the future of an antiviral drug in treating COVID-19 patients.

The visible light shows insignificant intrinsic absorption by nucleic acids and proteins without presence of chromophores. Unlike UV or gamma radiation, the visible light of electromagnetic spectrum does not initiate molecular ionisation. These properties could enable the use of USP lasers to selectively inactivate pathogens without harming desired biological constituents such as mammalian proteins in blood products. [142]. Therefore, the structure of a mammalian protein is well preserved [142]. Visible USP lasers have shown a broad-spectrum efficacy against both DNA and RNA viruses [142,143,144,145,146,147,148,149] including nonenveloped viruses that are conventionally difficult to inactivate.

### 4.4. Ultraviolet (UV) Therapy

The use of UV light as an antiviral agent has been the subject of much controversy. It is important to note that prolonged exposure to UVA or UVB or any use of UVC can be detrimental to health. The ultraviolet blood irradiation (UBI) was widely used to treat many diseases such as septicaemia, pneumonia and asthma. Its effects to treat infection are related to its immune-modulating therapy as well as its ability to normalise blood parameters. Low doses of UV can kill microorganisms by damaging the DNA, while any DNA repair enzymes can rapidly repair damage in the host cells [150]. However, its effectiveness still remains controversial due to the shallow penetration depth of the phonic energy. A study by Hashem et al., 2019 investigated the effect of Amotosalen/ UVA light in reducing the risk of MERS-CoV transmission, via human platelet concentrates, which minimises the risk of transfusion-related MERS-CoV transmission [151]. It was concluded that Amotosalen and UVA light was effective in reducing the contagion of MERS-CoV-spiked platelet concentrates and completely inactivated MERS-CoV by >4 logs.

## 5. Potential Future Scope of Phototherapy in Augmenting the COVID-19 Vaccine Production

Undoubtedly, vaccination is going to be the supreme cure for the currently circulating deadly SARS-CoV-2 strain. Global efforts in the field of research and diagnosis to fight this situation are unparalleled in terms of the rate and magnitude of vaccine production. As of today, there are more than a hundred potential candidate vaccines either; in the pre-clinical or developmental stage or under different phases of animal or human trials [152]. In spite of the escalation in speed for production and mass distribution, the vaccine must be strategically designed for safe and effective use. Imposing quality control measures on the methods of production and administration can greatly help in assuring high standards of public health safety [153].

### 5.1. Utilisation of USP Laser Irradiation for Inactivation of SARS-CoV-2 to Optimise Vaccine Production

Whole inactivated virus (WIV) vaccines are a rapid method to obtain vaccines of emerging viral strains [154]. This is because in comparison to other types of vaccines, WIV vaccines can be manufactured quickly by chemical or physical inactivation of a purified virus strain regardless of the need to identify antigens [155]. At the same time, they can produce an effective immune response. The traditional methods of pathogen inactivation methods are formalin, Beta-propiolactone (β- propiolactone), heat, ultraviolet light and gamma rays [154,155,156]. Recent report on the rapid development of an inactivated vaccine for SARS-CoV-2 infection indicated utilisation of β- propiolactone for virus inactivation [157]. However, the use of β-propiolactone or any other methods, as mentioned above, has been associated with several structural alterations in the virus proteins as well as the vaccine antigens leading to a suppressed immune response generation from the vaccine and an overall diminished potency [155,158]. Therefore, it is necessary to search for comprehensive techniques for safe and immunogenic WIV vaccine production.

During the last decade, the use of USP laser irradiation for preparation of WIV vaccines has been tried and tested to combat the influenza A virus subtype H1N1 [148,154,156]. Unlike its counterparts, USP irradiation utilises laser pulses with a pulse duration of several femtoseconds, which helps in the physical inactivation of the virus [154,156]. This occurs through the process of impulsive stimulated Raman scattering (ISRS), which causes rapid molecular vibrations within viral capsids through spontaneous and excited Raman scattering [142,148,159]. The resultant clumping of viral capsid proteins, which is a universal feature of viruses, leads to their inactivation. The use of visible light in the range of λ400 to λ700 nm, enables several characteristic features to this technique, such as (1) prevention of disintegration in the structure of the virus, thus reducing the risk of an adverse helper T cell, type 2 response caused by the vaccine; (2) prevention of a heat-initiated structural denaturation of B-cell epitopes; and (3) preserving the toll like receptor (TLR)-stimulating capacity of viral nucleic acids, thus upholding the potency of the virus which is sufficient to generate an effective immune response [154,156]. It has been proven that WIV vaccines can be inactivated by the chemical-free; therefore, the USP laser irradiation process could do so at a dose which is 10 times lower than that of the conventionally used formalin inactivation method required for the production of the HINI influenza vaccine [154]. In lieu of the beneficial effects of USP laser irradiation-mediated virus inactivation protocol, the use of the same could be a prospective method of choice for the production of the COVID-19 vaccine, provided all essential laboratory and clinical requirements and regulations are foreseen and met.

### 5.2. Potential Role of Lasers as COVID-19 Vaccine Adjuvants

Traditionally utilised vaccines possess poor immunogenic potential on their own and optimisation of their efficacy to treat infection holds a key role in the future of vaccine development [24]. Immunological or vaccine adjuvants are chemical compounds or macromolecules, which provide enhanced synergistic benefits to the vaccine antigen, resulting in improved and long-lasting immunological memory to combat a viral infection [24,160]. However, in spite of a higher rate of seroconversion in adjuvant vaccines, the latter are often supplanted by an increased likelihood of adverse local or systemic reactions, resulting in very few being commercially available while having Food and Drug administration (FDA) approval [160]. Some of the most commonly used vaccine adjuvants comprise particulate aluminium salts, alum with mono-phosphoryl lipid A (AS04) or squalene-based oil-in-water emulsion (AS03) [24,160,161]. The most common adverse effects noted with the use of the above-mentioned adjuvants are as follows; injection site reactions, reduced immunogenic potential with subsequent doses and inability to mediate a broad-spectrum immune response [24,160]. In a clinical study based in France, an intramuscular alum-adjuvanted vaccine injection was linked with the development of macrophagic myofascitis in some patients [162]. Additionally, in vitro analyses have shown that the use of an alum-complex vaccine adjuvant has several structural limitations, which degrade the potency of the vaccine [24,160,163,164]. A recently conducted pilot trial to test a vaccine for SARS-CoV-2, reports the utilisation of an alum vaccine adjuvant [157]. The precise demerits of the conventionally used adjuvants explained through the above-mentioned facts have instilled the urgency to discover novel, efficient and non-toxic vaccine adjuncts.

It is important to highlight that use of laser, as an adjunctive therapy to antiviral vaccines [laser vaccine adjuvants (LVA)] has captured the attention of many researchers. LVA are novel and efficient vaccine adjuvants, which are upcoming in the field of research and diagnosis for vaccine production [24,160]. When applied intra-muscularly LVA has demonstrated a boost in the Th1-mediated immune responses that are crucial to combat viral infections [165]. The ability of LVA to enhance the cell-mediated, in particular, the CD8+ T cell-mediated immune response, which is almost negligible by means of an alum vaccine adjuvant, has also been demonstrated [166]. LVA requires a transient photo-illumination with specific dosimetry at the intramuscular or intradermal sites [148]. This procedure avoids the formation of any kind of adjuvant-related complex unlike other vaccine adjuvants and therefore it is able to provoke the antigen presenting cells (APCs), without inducing any significant localised inflammatory response or foreign body tissue reactions [160]. LVA application shortens the pharmaceutical time-frame since they do not require any prior chemical preparation unlike other chemical compounds currently being utilised, as vaccines adjuvants [160,167,168]. LVA most certainly appears to be an efficient, simple, convenient and profitable treatment modality, without long-term adverse effects. Although, there exists ample evidence on the use of the LVA technique, in the past for viral infectious diseases, it is possible application in the management of COVID-19 is unexplored and needs to be established through laboratory and clinical analyses.

## 6. Conclusions

This review highlighted that PBMT can deactivate viruses and reduce viral load. This potential therapy could be a way forward via trans-tracheal or trans-dermal PBMT approach in the management COVID-19 patients. Equally, new innovative laser technologies have emerged such as LVAs and USP laser. The latter modality is well documented in the literature for its ability to selectively inactivate viruses by utilising femtosecond laser pulses. On the other hand, LEDs PBM of single or multiple wavelengths, delivered via clustered probe, can enhance immune responses and improve functionality of inflamed lungs. Nevertheless, utilisation of precise laser dosimetry and necessity to follow laser safety guidelines remains irrefutable. PDT is a well-documented modality in the literature for its effective photochemical reaction on eliminating the viability of SARS and MERS viruses in the blood, which ultimately eliminates the potential risk of CoVs transmission via blood products or its derivatives.

We answered our research question that the molecular and cellular mechanisms of action of phototherapy as a potential antioxidant treatment in enhancing immune response and reducing the host–viral interaction in patients infected with SARS-CoV-2. Therefore, it is a promising treatment modality which needs to be further validated for COVID-19 management by robust and rigorous randomised, double blind, placebo-controlled clinical trials to evaluate its impartial outcomes and safety.
